# Dahuang Zexie Decoction Protects against High-Fat Diet-Induced NAFLD by Modulating Gut Microbiota-Mediated Toll-Like Receptor 4 Signaling Activation and Loss of Intestinal Barrier

**DOI:** 10.1155/2017/2945803

**Published:** 2017-11-12

**Authors:** Jing Fang, Xiaoqi Sun, Boyu Xue, Nanyuan Fang, Min Zhou

**Affiliations:** ^1^The First College of Clinical Medicine, Nanjing University of Chinese Medicine, Nanjing 210023, China; ^2^Department of Police Tactics, Nanjing Forest Police College, Nanjing 210023, China; ^3^Department of Infectious Disease, Jiangsu Province Hospital of Traditional Chinese Medicine, Affiliated Hospital of Nanjing University of Chinese Medicine, Nanjing 210029, China

## Abstract

Increasing evidence suggests that intestinal dysbiosis, intestinal barrier dysfunction, and activated Toll-like receptor 4 (TLR4) signaling play key roles in the pathogenesis of NAFLD. Dahuang Zexie Decoction (DZD) has been verified to be effective for treating NAFLD, but the mechanisms remain unclear. In this study, we investigated the effects of DZD on NAFLD rats and determined whether such effects were associated with change of the gut microbiota, downregulated activity of the TLR4 signaling pathway, and increased expressions of tight junction (TJ) proteins in the gut. Male Sprague Dawley rats were fed high-fat diet (HFD) for 16 weeks to induce NAFLD and then given DZD intervention for 4 weeks. We found that DZD reduced body and liver weights of NAFLD rats, improved serum lipid levels and liver function parameters, and relieved NAFLD. We further found that DZD changed intestinal bacterial communities, inhibited the intestinal TLR4 signaling pathway, and restored the expressions of TJ proteins in the gut. Meanwhile ten potential components of DZD had been identified. These findings suggest that DZD may protects against NAFLD by modulating gut microbiota-mediated TLR4 signaling activation and loss of intestinal barrier. However, further studies are needed to clarify the mechanism by which DZD treats NAFLD.

## 1. Introduction

Nonalcoholic fatty liver disease (NAFLD), as the hepatic manifestation of metabolic syndrome [1], is typified by fat accumulation in the liver without significant alcohol consumption [2]. Recently, NAFLD has become the most common cause for chronic liver disease worldwide [3–5]. The prevalence rate of NAFLD is 25.24% globally, being highest in the Middle East and South America and lowest in Africa. NAFLD has been associated with obesity, type 2 diabetes mellitus, hyperlipidemia, hypertension, fibrosis, and hepatocellular carcinoma [6]. However, the pathogenesis of NAFLD has not been completely clarified. It is well-established that the gut microbiota is significantly involved in the pathogenesis of NAFLD [7, 8]. The gut is open to the outer environment, and the gut microbiota is a complex microbial community inhabiting the intestinal tract that includes 100 trillion bacteria with over 1000 species. It contains genetic materials severalfold those of human genome and produces considerable metabolites and peptides. Accumulating evidence has proven that NAFLD increased energy harvesting upon intestinal dysbiosis or bacterial overgrowth [9, 10]. Patients with NAFLD have lower percentages of Bacteroidetes and Ruminococcaceae than those in normal subjects [11, 12]. Besides, damage of the intestinal barrier also significantly contributes to NAFLD. The intestinal mucosal mechanical barrier is the first defense of intestinal barrier, which is composed of intestinal epithelial cells (IECs), tight junction (TJ), and the mucous layer that covers the surface of IECs. TJ plays a crucial role in maintaining the integrity of the intestinal barrier, which promotes nutrient and water transport and also protects against gut-derived pathogens [13]. In the case of NAFLD, intestinal bacterial overgrowth generates gut-derived pathogens such as endotoxin or lipopolysaccharide (LPS) which are the main components of cell walls in Gram-negative bacteria. Meanwhile, they can activate the Toll-like receptor 4 (TLR4) signaling pathway. After binding TLR4 on the cell membrane, they increase the intestinal permeability by downregulating TJ proteins. As we all know, the liver is located in the proximity of the gut, receiving 75% of its blood supply through the portal vein. The portal vein flow not only carries nutrients, but also translocates microbial products and bacteria. Due to increased intestinal permeability (leaky gut), gut-derived pathogens can penetrate the intestinal barrier into the portal vein and cause NAFLD by inducing liver inflammation and fat deposition.

In Western countries, there is still no proven medical therapy for NAFLD hitherto. In contrast, traditional Chinese medicine drugs have exhibited remarkable therapeutic effects on NAFLD. Dahuang Zexie Decoction (DZD), as a Chinese herbal formula, consists of three herbs, Zexie* (Alisma orientale)*, Baizhu* (Atractylodes macrocephala)*, and Dahuang* (Rheum palmatum)*. It was developed from Zexie Decoction, a classical prescription documented in Synopsis of the Golden Chamber, which was completed in the Chinese Han dynasty (206 BC-220 AD). Zexie Decoction only has two herbs, Zexie* (A. orientale)* and Baizhu* (A. macrocephala)*. It can obviously mitigate NAFLD and decrease blood lipid levels [14]. Thereby motivated, we established a rat model of high-fat diet- (HFD-) induced NAFLD and then evaluated the effects of DZD on the liver function and gut microbiota, together with the expressions of intestinal TJ proteins and members in the TL4 signaling pathway in these rats.

## 2. Materials and Methods

### 2.1. Preparation of DZD

We prepared DZD by a mixture comprising the following three dried herbs: Zexie (*Alisma orientalis*, 30 g), Baizhu (*Atractylodes macrocephala*, 12 g), and Dahuang (*Rheum palmatum*, 15 g). The herbs were extracted with water, concentrated to the density of 1 g crude herb/ml, and stored at −20°C until further use. All the herbal components were purchased from Baicaotang Outpatient Department, Nanjing University of Chinese Medicine (Nanjing, China). Herbs were obtained from qualified suppliers on the basis of standards specified in the Chinese Pharmacopoeia (2010 Edition).

### 2.2. Animals

The protocols for animal studies were reviewed and approved by the Animal Studies Ethics Committee of Nanjing University of Chinese Medicine. Eight-week-old male Sprague Dawley (SD) rats (*n* = 24) were housed in a controlled environment (12 h–12 h light–dark cycle) in the Animal Center of Nanjing University of Chinese Medicine. After one week of acclimation on normal diet, the rats were randomly divided into three groups and fed either normal diet or HFD. Normal diet (fat contributed 10% calories) and HFD (fat contributed 45% calories) were purchased from Jiangsu Medicine Biological & Pharmaceutical Co., Ltd. (Yangzhou, China). A control group was fed normal diet for 16 weeks (*n* = 8), and the remaining sixteen rats were fed HFD for 16 weeks. After 16 weeks of HFD feeding, the sixteen rats were further divided into 2 groups: NAFLD group and DZD group (*n* = 8). The DZD group received daily gavage of 5.13 g/kg DZD (density: 1 g crude herb/ml) in addition to HFD for four weeks, while the NAFLD group was gavaged with the same volume of saline for four weeks. The control group was fed normal diet and saline for four weeks. Body weight gain was assessed once a week. After 4 weeks of gavage, the animals were anesthetized with 4% chloral hydrate and then sacrificed. Blood was drawn and collected into tubes. Liver tissue was weighed and collected. Intestine and feces samples were also collected.

### 2.3. Reagents

Antibody against TLR4 was purchased from Abnova (TW, China). Antibodies against myeloid differentiation factor 88 (MyD88),* p*-JNK, JNK,* p*-ERK, and ERK were purchased from Cell Signaling Technology (MA, USA). Antibody against occludin and goat anti-mouse secondary antibody were purchased from Abcam (MA, USA). Antibody against zonula occludens-1 (ZO-1) was purchased from Thermo Fisher Scientific (MA, USA). Antibody against *β*-actin and goat anti-rabbit secondary antibody were purchased from Santa Cruz Biotechnology (CA, USA).

### 2.4. Biochemical Assays

Triglyceride (TG), total cholesterol (TC), low density lipoprotein-cholesterol (LDL-C), high density lipoprotein-cholesterol (HDL-C), alanine aminotransferase (ALT), and aspartate aminotransferase (AST) in serum were measured by commercial kits (Nanjing Jiancheng Institute of Biotechnology, Nanjing, China) according to the manufacturer's instructions.

### 2.5. Histopathological Examination

Liver pieces of about 5 mm in all dimensions were obtained from rats and fixed in 4% formaldehyde for 15 min. Afterwards, the specimens were sequentially equilibrated in 30% sucrose, 15% sucrose/50% optimal cutting temperature medium (OCT, Sakura Finetek, Torrance, CA, USA), and 100% OCT. Liver pieces were subsequently frozen in OCT and 10 *μ*m-thick sections were cut with a cryostat. The sections were then stained with hematoxylin/eosin (HE) and visualized by light microscopy.

### 2.6. Immunohistochemical Assay

Ileum and colon tissues were obtained to detect ZO-1 and occludin. The above sections were used for immunohistochemistry, and rehydrated before immunostaining. After blocking, the sections were incubated with rabbit anti-rat ZO-1 and occludin antibodies (Invitrogen, USA). The immunostaining results were reviewed and scored independently by two pathologists.

### 2.7. Western Blotting

The protein expressions of ZO-1, occludin, TLR4, MyD88,* p*-JNK, JNK,* p*-ERK, and ERK in the gut were detected by Western blotting. In brief, total protein was extracted from distal ileum and colon tissues, and the concentrations of supernatants were measured using the BCA protein assay (Thermo Fisher). Aliquots of supernatants containing 30 *μ*g of protein were electrophoresed on 10% (w/v) sodium dodecyl sulfate-polyacrylamide gels and transferred onto polyvinylidene difluoride membranes. After blocking for 1 h with 5% (w/v) skimmed milk, the membranes were incubated with primary antibodies at 4°C overnight and then washed by tris-buffered saline and incubated with secondary antibodies for 1 h. Protein bands were analyzed with image analysis software Quantity one. The results were expressed as ratios relative to *β*-actin as the internal control.

### 2.8. DNA Extraction, 16S Ribosomal RNA (rRNA) Gene Sequencing, and Microbial Analysis of Fecal Samples

Fecal samples were collected and frozen at −80°C within 3 h after sampling. DNA extraction was performed using a QIAamp Fast DNA Stool Mini kit (Qiagen, California, USA). Purity was determined and concentration was calculated. The V3 region of bacterial 16S rRNA gene was amplified by PCR. The bacterial genomic DNA was amplified by PCR with forward primer (5′-TCGTCGGCAGCGTCAGATGTGTATAAGAGACAGCCTACGGGNGGCWGCAG) and reverse primer (5′-GTCTCGTGGGCTCGGAGATGTGTATAAGAGACAGGACTACHVGGGTATCTAATCC) for the V3 hypervariable region. Purified amplicons were pooled into equimolar concentrations, and paired-end sequencing was performed using an Illumina MiSeq instrument (Illumina, San Diego, California, USA). The representative sequences of OTUs were used to analyze *α*-diversity (Chao index and Shannon diversity index) on the basis of their relative abundances. A heatmap was generated according to the relative abundances of OTUs by R software (https://www.R-project.org). Phylogenetic *β*-diversity measures such as unweighted UniFrac significance test, principal coordinate analysis, and nonmetric multidimensional scaling were performed using the representative sequences of OTUs for each sample by the Mothur program to analyze community and phylogenesis. Taxonomy-based analyses were performed for taxonomic classification using the ribosomal database project classifier with a 60% bootstrap score.

### 2.9. High-Performance Liquid Chromatography Coupled with Mass Spectrometry (HPLC-MS)

DZD water extract was analyzed by high-performance liquid chromatography with hybrid linear ion trap Orbitrap mass spectrometry (HPLC-LTQ/Orbitrap) (Thermo Fisher Scientific, USA) with an Accucore C chromatographic column (182.1 mm × 150 mm, 0.26 *μ*m, Thermo Fisher Scientific, USA) and a 5 cm Lot 13375 guard column (Thermo Fisher Scientific, USA). The mobile phase consisted of (A) 100% acetonitrile and (B) 100% water with 0.1% formic acid. Gradient elution was conducted by using 5%–12% (0–30 min), 40% (35 min), 40% (52 min), 70% (63 min), 85% (67 min), and 85% acetonitrile (70 min). The flow rate was 1 mL/min and the detection range was full wavelength. The mass spectrometry (MS) method consisted of positive and negative ion detection model. The ESI source parameters were set as follows: ion spray voltage 2.4 kV, capillary temperature 300°C, source heater temperature 110°C, sheath gas (N2) 40 arbitrary units, auxiliary gas (N2) 10 arbitrary units, and sweep gas (N2) 0 arbitrary units. The Orbitrap analyzer scanned the mass with the range of *m*/*z* 120–900.

### 2.10. Statistical Analysis

SPSS 18.0 and GraphPad Prism 5 were used for statistical analysis. Data were expressed as mean ± standard error of mean (SEM). One-way analysis of variance with Tukey's correction was applied for differences between two groups, and *P* < 0.05 was accepted as statistically significant.

## 3. Results

**Figure 1:**
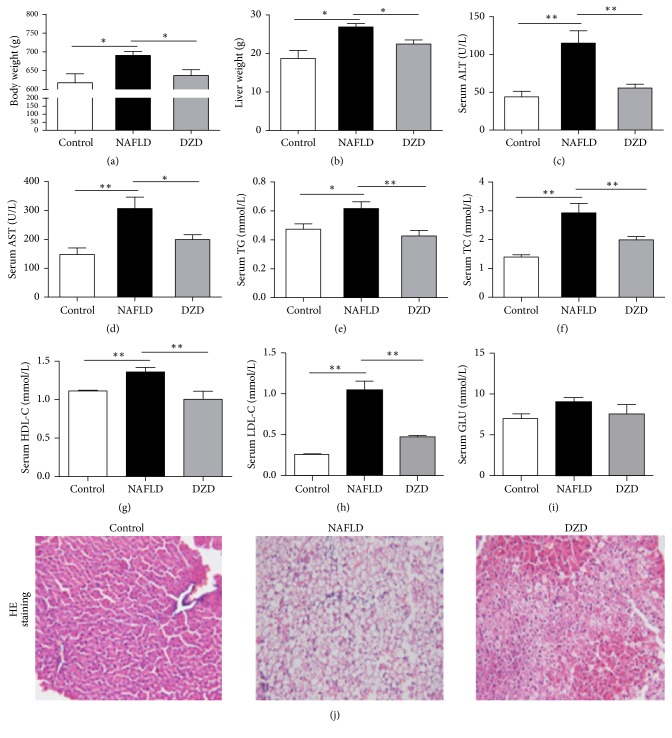
*Therapeutic effects of DZD on rat model of HFD-induced NAFLD*. Male SD rats (8 weeks old) were fed HFD for 16 weeks, followed by either 4-week DZD feeding or normal saline supplementation through gavage, while the rats fed normal diet were set as controls. The rats were sacrificed, from which the liver and serum were collected. Body weight (a), liver weight (b), serum ALT (c), serum AST (d), serum TG (e), serum TC (f), serum HDL-C (g), serum LDL-C (h), and serum GLU (i) levels were detected. Liver sections were stained with HE (j). The data are expressed as mean ± SEM. *N* = 5 to 8 for every group. ^*∗*^0.01 < *P* < 0.05; ^*∗∗*^*P* < 0.01. ALT: alanine aminotransferase; AST: aspartate transaminase; TG: triglyceride; TC: total cholesterol; HDL-C: high density lipoprotein-cholesterol; LDL-C: low density lipoprotein-cholesterol; GLU: glucose; HE: hematoxylin/eosin.

*(1) Therapeutic Effect of DZD on Rat Model of HFD-Induced NAFLD*. To evaluate the therapeutic effect of DZD on NAFLD in the HFD-fed rat model, we detected the body weight, liver weight, serum lipid levels, liver function parameters, and pathological changes of three groups. Compared to the control group, the HFD group had higher body weight, liver weight, and serum ALT, AST, TG, TC, HDL-C, and LDL-C levels (Figures 1(a)–1(h)). DZD treatment for 4 weeks significantly decreased body and liver weights and restored serum lipid levels and liver function parameters compared with those of the HFD group (Figures 1(a)–1(h)). Meanwhile, the body weight, liver weight, serum lipids, and liver function parameters of the DZD group were similar to those of the control group. The three groups also had similar serum glucose levels (Figure 1(i)). Consistently, the liver sections of the HFD group showed extensive macrosteatosis and hepatocyte ballooning which were relieved in those of DZD-treated rats (Figure 1(j)). Collectively, DZD exerted beneficial effects on the NAFLD rat model.

**Figure 2:**
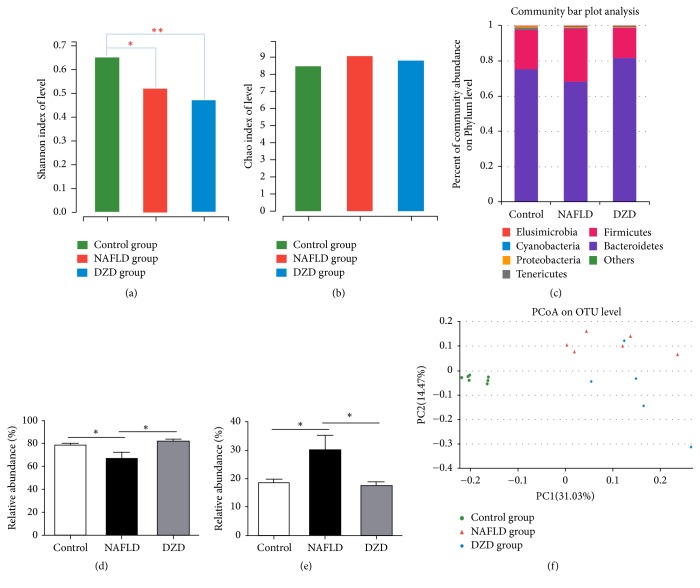
*Effects of DZD therapy on composition of gut microbiota*. Bacterial diversity is shown by the Shannon index (a). Bacterial richness is shown by the Chao index (b). Species on the phylum level (c), as well as alterations of Bacteroidetes (d) and Firmicutes (e) are indicated. PCoA score plot based on the unweighted significance test (f). The data are expressed as mean ± SEM. *N* = 5 to 8 for every group. ^*∗*^0.01 < *P* < 0.05; ^*∗∗*^*P* < 0.01.

**Figure 3:**
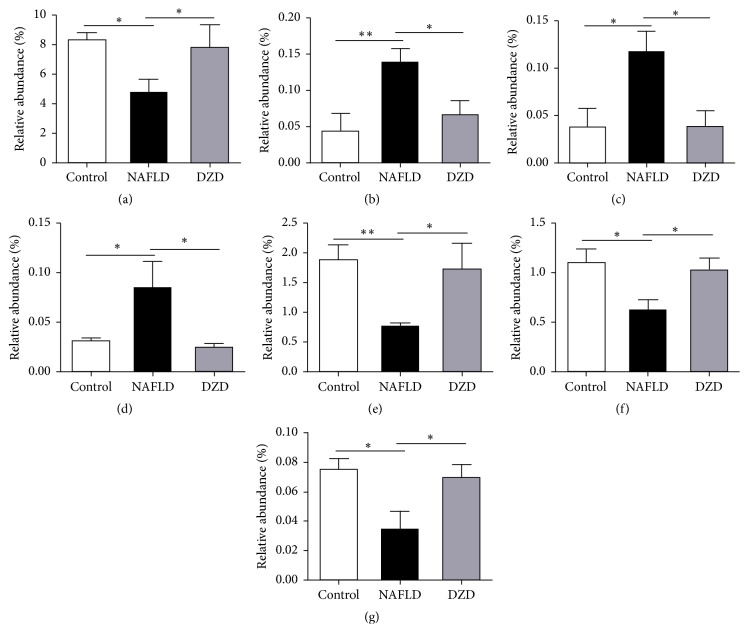
*Relative abundances of gut microbiota on different taxonomic levels among three groups*. On the family level, the alterations of Ruminococcaceae (a) and Desulfovibrionaceae (b) were indicated. At the genus level, the alterations of* Desulfovibrio* (c),* Escherichia*/*Shigella* (d),* Bacteroides* (e),* Oscillibacter* (f), and* Butyricicoccus* (g) were indicated. The data are expressed as mean ± SEM. *N* = 5 to 8 for every group. ^*∗*^0.01 < *P* < 0.05; ^*∗∗*^*P* < 0.01.

*(2) Effects of DZD Therapy on the Gut Microbiota Composition of NAFLD Rats*. NAFLD is associated with intestinal dysbiosis, so we analyzed the effects of HDF and DZD on the gut microbiota composition. We used MiSeq technology to conduct bacterial 16S rRNA sequencing after 4 weeks of treatment. The quality of sequencing, which included microbial richness and biodiversity, met the requirements for subsequent analysis. Both HFD and DZD decreased the Shannon index compared with that of the control group (Figure 2(a)), so they significantly reduced the diversity of gut microbiota. Meanwhile, the three groups had similar richness, as indicated by the Chao index (Figure 2(b)). Samples from the three groups formed distinct clusters in the ordination plot, suggesting that HFD and DZD induced main changes in the gut microbiota (Figure 2(c)). The HFD group had a higher relative abundance of Firmicutes and a lower relative abundance of Bacteroidetes than those of the control group, and DZD decreased the proportion of Firmicutes and increased that of Bacteroidetes on the phylum level (Figures 2(d) and 2(e)). Besides, principle component analysis (PCoA) was performed to compare the differences in bacterial communities between different groups on the OTU level (Figure 2(f)). In addition, changes of intestinal microbiota on the family level showed that Ruminococcaceae significantly decreased and Desulfovibrionaceae significantly increased in the HFD group compared to those in the control group (Figures 3(a) and 3(b)). In contrast, DZD administration restored these proportions (Figures 3(a) and 3(b)). On the genus level, the relative abundances of* Desulfovibrio *and* Escherichia/Shigella* were significantly higher in the NAFLD group, which were reversed by DZD intervention (Figures 3(c) and 3(d)). Meanwhile, the relative abundances of* Bacteroides*,* Oscillibacter*, and* Butyricicoccus* were significantly lower in the NAFLD group, which were restored by DZD intervention (Figures 3(e)–3(g)). In short, DZD altered the intestinal microbiota composition of NAFLD rats.

**Figure 4:**
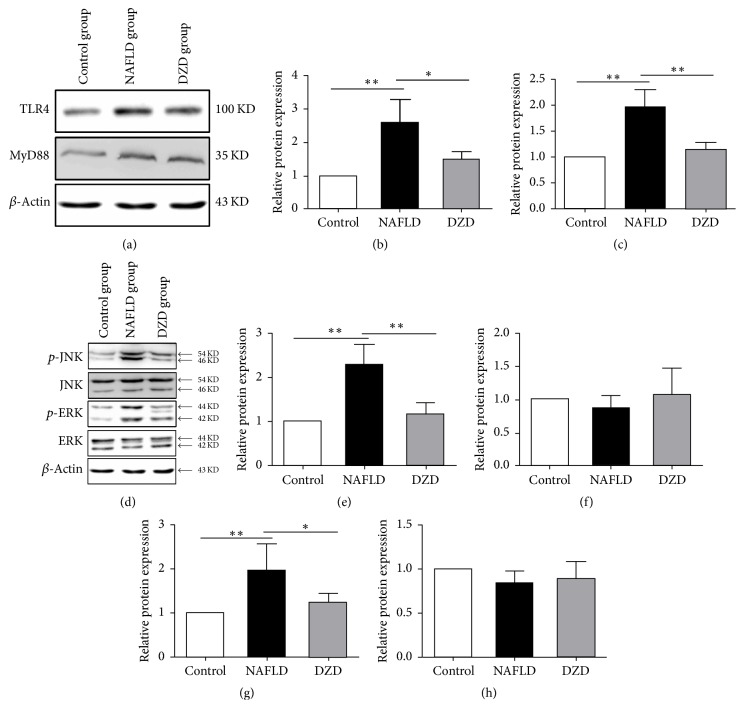
*DZD intervention activated the TLR4 signaling pathway of the ileum*. The ilea of rats were collected. TLR4, MyD88 (a),* p*-JNK, JNK,* p*-ERK, and ERK (d) protein expressions of ileum tissues were analyzed by Western blot. Bar graphs are the plots of TLR4 (b), MyD88 (c),* p*-JNK (e), JNK (f),* p*-ERK (g), and ERK (h). The data are expressed as mean ± SEM. *N* = 5 to 8 for every group. ^*∗*^0.01 < *P* < 0.05; ^*∗∗*^*P* < 0.01.

*(3) DZD Intervention Activated the TLR4 Signaling Pathway in the Ileum*. Damage of the intestinal barrier may induce intestinal endotoxemia and subsequent TLR4 activation in the liver, which is implicated in the pathogenesis of NAFLD [15, 16]. Kim et al. reported that HFD-induced intestinal inflammatory response by activating the TLR4 signaling pathway [17]. To further investigate whether DZD regulated intestinal inflammation via this pathway, the expressions of TLR4 and mitogen-activated protein kinase (MAPK) members in the ileum were detected by Western blotting. The protein expression levels of TLR4 and MyD88 were upregulated in the ileum of the HFD group (Figures 4(a)–4(c)). After treatment with DZD, such levels were restored to normal (Figures 4(a)–4(c)). The phosphorylation levels of ERK and JNK in the HFD group exceeded those of the control group (Figures 4(d), 4(e), and 4(g)). However, the three groups had similar ERK and JNK levels (Figures 4(d), 4(f), and 4(h)). Similarly, DZD downregulated the phosphorylation levels of these proteins (Figures 4(d), 4(e), and 4(g)). Thus, activation of the TLR4 signaling pathway in the ileum was enhanced by HFD, whereas DZD reduced the activity of this pathway to normal.

**Figure 5:**
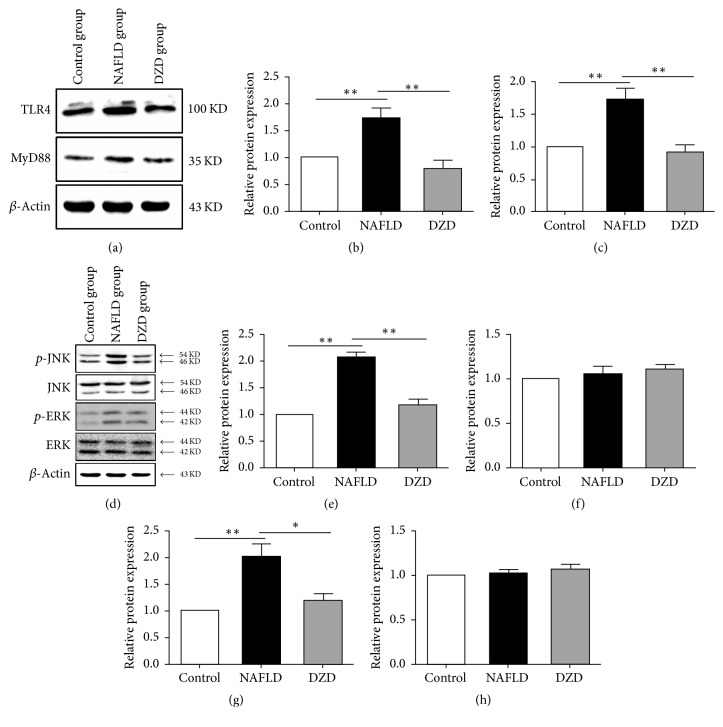
*DZD intervention activated the TLR4 signaling pathway of the colon*. The colons of rats were collected. TLR4, MyD88 (a),* p*-JNK, JNK,* p*-ERK, and ERK (d) protein expressions of colon tissues were analyzed by Western blot. Bar graphs are the plots of TLR4 (b), MyD88 (c),* p*-JNK (e), JNK (f),* p*-ERK (g), and ERK (h). The data are expressed as mean ± SEM. *N* = 5 to 8 for every group. ^*∗*^0.01 < *P* < 0.05; ^*∗∗*^*P* < 0.01.

*(4) DZD Intervention Activated the TLR4 Signaling Pathway in the Colon*. We also detected the protein expression levels of members in the TLR4 signaling pathway in the colon of different groups. HFD upregulated the expression levels of TLR4 and MyD88 in the colon (Figures 5(a)–5(c)). Compared to the HFD group, DZD intervention downregulated their protein expressions (Figures 5(a)–5(c)). Nevertheless, there was no significant difference between control and DZD groups. Identical to results in the ileum, the phosphorylation levels of ERK and JNK in the HFD group were higher than those in the control group, which were downregulated by DZD (Figures 5(d), 5(e), and 5(g)). Similarly, the total protein expression levels of ERK and JNK in the colon were not significantly different among the three groups (Figures 5(d), 5(f), and 5(h)). Taken together, HDF caused activation of the TLR4 signaling pathway in the colon, which was attenuated by DZD.

**Figure 6:**
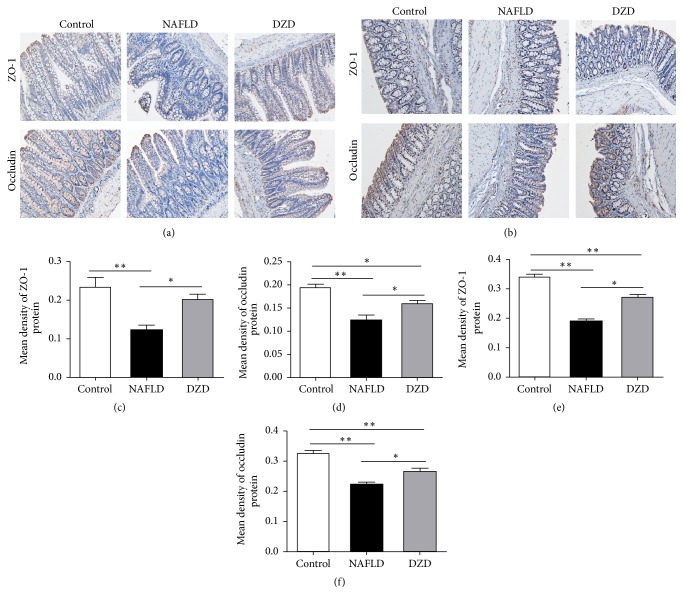
*DZD intervention alleviated loss of intestinal TJ proteins*. Immunohistochemical staining for ZO-1 and occludin in ileum (a) and colon (b) tissues. Bar graphs are the plots of ZO-1 (c) and occludin (d) in the ileum and ZO-1 (e) and occludin (f) in the colon. The data are expressed as mean ± SEM. *N* = 5 to 8 for every group. ^*∗*^0.01 < *P* < 0.05; ^*∗∗*^*P* < 0.01.

*(5) DZD Intervention Relieved Loss of Intestinal Barrier Integrity in the NAFLD Model*. TJ proteins of the intestinal mucosa, such as ZO-1 and occludin, are crucial to maintenance of the intestinal barrier [18]. Decrease in the expressions of ZO-1 and occludin can increase the intestinal permeability and play an important role in the pathophysiology of NAFLD [19]. To assess the effects of HFD and DZD on the intestinal barrier function, we used immunohistochemistry to detect the protein expression levels of ZO-1 and occludin in the gut. In accordance with previous studies, ZO-1 and occludin were highly expressed in both the ileum and colon of the control group, which were reduced by HFD intervention (Figures 6(a)–6(f)). After treatment with DZD, the protein expressions of ZO-1 and occludin in the gut were recovered compared with those of the HFD group (Figures 6(a)–6(f)). Therefore, the expressions of ZO-1 and occludin in the gut, which were reduced by HDF, were restored by DZD, thereby mitigating the loss of intestinal barrier integrity in the NAFLD model.

**Figure 7:**
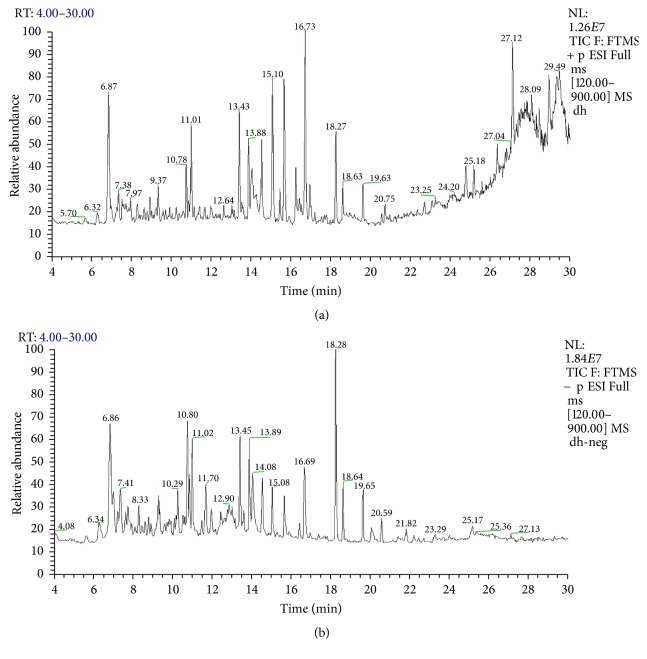
HPLC chromatogram of DZD. Total ions chromatograms of DZD in positive (a) and negative (b) ion modes. Identification of potential constituents of DZD (see Table 1).

*(6) Identification of Components of DZD*. The components of DZD water extract were identified by HPLC-MS. Ten potential compounds, that is, gallic acid, chrysophanol, rhein, emodin, physcion, alisol C monoacetate, alisol B, atractylenolide I, atractylenolide II, and atractylenolide III, were identified (Figure 7, Table 1). The characterizations and sources of these compounds are listed in Table 1.

## 4. Discussion

NAFLD is the liver manifestation of metabolic syndrome, with a high incidence rate worldwide [20, 21]. At present, NAFLD has become the main cause for hepatocellular carcinoma in the United States [22, 23]. There are still no effective therapies for NAFLD patients in Western countries [24]. Contrarily, the therapeutic effects of traditional Chinese medical formulations on NAFLD have been well-documented. Our study firstly demonstrated that DZD mitigated HFD-induced NAFLD. Sixteen weeks of HFD feeding increased body weight, as well as disturbing liver function parameters and blood lipid levels (Figures 1(a)–1(h)). Liver histological examination revealed extensive macrosteatosis and hepatocyte ballooning (Figure 1(j)), being consistent with previous studies. We also demonstrated that DZD administration for 4 weeks reduced body weight and blood lipid levels, improved liver function parameters, and alleviated the liver pathological changes of NAFLD rats (Figures 1(a)–1(h) and 1(j)). Hence, DZD indeed relieved HFD-induced NAFLD.

In sequencing studies, the microbiota composition of NAFLD patients markedly changed after HFD feeding, so DZD may mitigate NAFLD by altering the gut microbiota following oral administration. Firstly, we assessed the effect of DZD on the gut microbiota by using multivariate analysis. The Shannon index and Chao index represented the diversity and richness of gut microbiota in each sample, respectively. HFD and DZD both significantly reduced the diversity of gut microbiota compared with that of the control group (Figure 2(a)). However, the three groups had similar richness (Figure 2(b)). Subsequently, we detected the gut microbiotas in different groups on the phylum level. The NAFLD group had lower percentage of Bacteroidetes but higher percentage of Firmicutes (Figures 2(c)–2(e)), similar to previous studies [25–27]. Nevertheless, the results were restored by DZD treatment (Figures 2(c)–2(e)).

The serum levels of LPS, as a gut bacteria-derived product, are elevated in NAFLD patients. LPS is an important component of the outer membrane of Gram-negative bacteria of which Bacteroidetes is the main type. In our study, Bacteroidetes decreased in the NAFLD group, but damage of the intestinal barrier and increase of the gut permeability induced more gut-derived bacterial LPS to enter blood via the hepatic portal system [28]. The integrity of the intestinal barrier is closely related to gut bacteria-derived products, and LPS can increase the gut permeability by inducing intestinal inflammatory response. Therefore, we further tested the relative abundances of Gram-negative bacteria on family and genus levels.* Bacteroides*, a genus of Bacteroidetes, changed following the same trend. Interestingly, the fecal microbiome of the NAFLD group had increased Desulfovibrionaceae family,* Desulfovibrio *genus, and* Escherichia/Shigella *genus (Figures 3(b)–3(d)). The relative abundances of Desulfovibrionaceae, a family of sulfate-reducing bacteria (SRB), and* Desulfovibrio*, a genus of SRB, increased in the NAFLD group, which were reversed by DZD (Figures 3(b) and 3(c)). A previous study showed that diet increased SRB* in vitro* [29]. These gut bacteria can release hydrogen sulfide which is a genotoxic gas that affects epithelial intestinal cell integrity and causes barrier dysfunction [30]. Meanwhile, Lodowska et al. verified the high biological activity of LPS from* Desulfovibrio* [31]. Although these bacteria have low contents, LPS in them can evidently induce inflammation. Herein,* Escherichia/Shigella* genus was significantly enriched in the NAFLD group compared to that in the control group, which decreased to normal levels after DZD treatment (Figure 3(d)). Increase of* Escherichia/Shigella *can induce intestinal inflammation, causing intestinal barrier dysfunction [32, 33]. Therefore, even a small number of Gram-negative bacteria can still destroy the intestinal barrier through intense proinflammatory effects.

In our study, the fecal microbiome of the NAFLD group had decreased Ruminococcaceae family,* Oscillibacter *genus, and* Butyricicoccus *genus (Figures 3(a), 3(f), and 3(g)), but the results were restored by DZD treatment. Jiang et al. demonstrated that the Ruminococcaceae family and the* Oscillibacter* genus increased significantly in the healthy group compared to those in the NAFLD group [34]. Ruminococcaceae,* Oscillibacter*, and* Butyricicoccus* are short chain fatty acid- (SCFA-) producing bacteria which prevent metabolic endotoxemia by strengthening the gut barrier [35–37]. SCFAs can protect against gut inflammation and decrease intestinal permeability [38, 39]. Changes of these gut bacteria induce decrease of SCFAs, increasing the intestinal permeability. Nevertheless, we herein proved that DZD invention restored the changes of these gut bacteria induced by HFD.

In this study, we focused on the influence of gut microbiota-mediated inflammation activation on the intestinal barrier, so we detected the changes of inflammatory signaling pathway in the gut. LPS is a special pathogen-associated molecular pattern and one of microbial products, being able to activate inflammatory pathways by binding TLR4. As a result, intestinal inflammatory response then occurs [40]. Studies have suggested that TLR4-mediated signaling of the gut potently drove the progression of NAFLD [41, 42]. As the receptor of LPS, TLR4 is expressed on the membranes of hepatocytes, IECs, immune cells, and so on. MyD88 is a downstream adaptor protein for all TLRs, except for TLR3 [43]. The TLR4-MyD88-MAPKs signaling cascade is crucial to inflammatory response and NAFLD progression. The MAPK signaling pathway is involved in a variety of physiological and pathological processes such as cell growth, inflammation, apoptosis, and proliferation [44–46]. ERK MAPK and JNK MAPK are two main components of the MAPK pathway. Activating TLR4 in the gut induces the inflammation of intestinal mucosa by mediating the phosphorylation of ERK and JNK. To further investigate whether DZD can regulate intestinal inflammation via the TLR4 signaling pathway, the expressions of members in the TLR4 signaling pathway of the ileum and colon were detected by Western blotting. The protein expression levels of TLR4 and MyD88 in the ileum were upregulated in the HFD group (Figures 4(a)–4(c)). After treatment with DZD, such levels were restored to normal (Figures 4(a)–4(c)). The total protein levels of ERK and JNK were similar among the three groups (Figures 4(d), 4(f), and 4(h)). However, the phosphorylation levels of these two proteins in the HFD group surpassed those of the control group (Figures 4(d), 4(e), and 4(g)). DZD downregulated the phosphorylation levels of ERK and JNK in NAFLD rats (Figures 4(d), 4(e), and 4(g)). These results confirmed that activation of the TLR4 signaling pathway in the ileum was upregulated by HFD. Meanwhile, DZD recovered the activity of the TLR4 signaling pathway to normal. Similar results were observed in the colon (Figures 5(a)–5(h)). Taken together, activation of TLR4 signaling in both the ileum and colon, which was induced by HDF, was downregulated by DZD.

Microbiota participates in liver diseases largely via the inflammatory pathway triggered by the interactions between intestinal bacteria and the intestinal barrier. The increased intestinal permeability in NAFLD patients has been attributed to intestinal microbiota [47]. Additionally, the gut microbiota influences the intestinal barrier function, while dysfunction of this barrier and LPS is closely related in human and animal models [48, 49]. A former research reported that LPS significantly induced the downregulation and redistribution of TJ proteins in Caco2 monolayers, as well as promoting the increase of intestinal epithelial permeability [50]. Damage of the intestinal barrier may result in translocation of intestinal bacteria and then entrance of gut-derived pathogens into portal circulation through the highly permeable intestinal barrier, triggering NAFLD eventually [51–53]. TJ proteins suppress the paracellular permeability and thus contribute essentially to the intestinal barrier, as a defense line, thereby impeding the entrance of intestinal pathogens into the liver. Intestinal permeability is regulated by TJ proteins of which transmembrane proteins ZO-1 and occludin have attracted wide attention. Given that ZO-1 and occludin are of great significance to the integrity of TJs, their downregulation is responsible for the disruption of TJ structure and the increase in paracellular permeability [54, 55]. In this study, immunohistochemistry revealed that HFD deceased the expressions of ZO-1 and occludin in both the ileum and colon (Figures 6(a)–6(f)). Accordingly, DZD upregulated such expressions (Figures 6(a)–6(f)) and then boosted the intestinal barrier function.

Furthermore, we analyzed the main components of DZD by using high-performance liquid chromatography coupled with mass spectrometry and successfully identified ten potential components of DZD, that is, gallic acid, chrysophanol, rhein, emodin, physcion, alisol C monoacetate, alisol B, atractylenolide I, atractylenolide II, and atractylenolide III (Figure 7, Table 1). Huang et al. demonstrated that gallic acid could improve high-fat diet- (HFD-) induced dyslipidaemia and hepatosteatosis [56]. Meng et al. reported that alisol B protected against nonalcoholic steatohepatitis in mice by activating farnesoid X receptor [57]. Rhein and emodin also can improve NAFLD induced by HFD [58, 59], and emodin can restore the increased intestinal permeability by inhibiting inflammation response [60]. Meanwhile, a former research revealed that alisol C monoacetate and alisol B could inhibit LPS-induced inflammatory reaction [61]. Although it is unclear whether atractylenolide can improve NAFLD, it has a definite anti-inflammatory and antiapoptosis effect [62, 63]. Due to complicated components of Chinese herbal medicine, we only identified ten of the main components of DZD. Further studies are still needed to clarify the main components of DZD and the potential mechanism for the therapeutic effects of DZD on NAFLD.

In conclusion, intestinal dysbacteriosis, activation of the TLR4 signaling pathway in the gut, and intestinal barrier dysfunction played important roles in NAFLD. Moreover, DZD changed intestinal bacterial communities, inhibited the intestinal TLR4 signaling pathway, restored the expressions of TJ proteins in the gut, and finally relieved HFD-induced NAFLD. Gallic acid, chrysophanol, rhein, emodin, physcion, alisol C monoacetate, alisol B, atractylenolide I, atractylenolide II, and atractylenolide III were ten main components of DZD. However, further studies are still needed to unravel the mechanism by which DZD treats NAFLD.

## Figures and Tables

**Table 1:** Identification of potential components of DZD.

Number	Rt (min)	Molecular formula	Molecular weight (*m*/*z*)	Observed ion peaks (*m*/*z*)	Identification
(1)	1.05	C7H6O5	170.12	169[M−H]^−^, 125	Gallic acid
(2)	13.56	C15H10O4	254.23	254[M+H]^+^, 237	Chrysophanol
(3)	14.08	C15H8O6	284.22	283[M−H]^−^, 239	Rhein
(4)	16.67	C15H10O5	270.24	269[M−H]^−^, 240.9, 225, 180.9	Emodin
(5)	14.31	C16H12O5	284.27	283[M−H]^−^, 268	Physcion
(6)	14.62	C32H48O6	528.728	529[M+H]^+^, 511, 469, 451, 415	Alisol C monoacetate
(7)	18.31	C30H48O4	472.70	473[M+H]^+^, 455, 437, 383, 365	Alisol B
(8)	15.46	C15H18O2	230.30	231[M+H]^+^, 213, 203, 185, 157, 143	Atractylenolide I
(9)	17.79	C15H20O2	232.32	233[M+H]^+^, 215, 197, 187, 159, 151	Atractylenolide II
(10)	15.47	C15H20O3	248.32	249[M+H]^+^, 231, 203, 189, 163, 135.69	Atractylenolide III
